# Personality Traits and Mental Health among Lebanese Medical Students: The Mediating Role of Emotional Intelligence

**DOI:** 10.3390/healthcare10122516

**Published:** 2022-12-12

**Authors:** Elsa Sfeir, Radwan El Othman, Muna Barakat, Souheil Hallit, Sahar Obeid

**Affiliations:** 1 School of Medicine and Medical Sciences, Holy Spirit University of Kaslik, Jounieh P.O. Box 446, Lebanon; 2 Department of Internal Medicine, American University of Beirut Medical Center, Beirut 1107 2020, Lebanon; 3 Department of Clinical Pharmacy & Therapeutics, School of Pharmacy, Applied Science Private University, Amman 11931, Jordan; 4 Research Department, Psychiatric Hospital of the Cross, Jal Eddib P.O. Box 60096, Lebanon; 5 Social and Education Sciences Department, School of Arts and Sciences, Lebanese American University, Jbeil 1401, Lebanon

**Keywords:** personality traits, extraversion, neuroticism, openness to experience, agreeableness, conscientiousness, emotional intelligence, depression, anxiety, stress, medical students

## Abstract

Medical students face daily challenges such as large workload, time commitment and clinical environment pressure leading to a higher risk of psychological distress. The aim of our study was to assess the relationship between personality traits and depression, anxiety, and stress among Lebanese medical students and to evaluate the mediating role of emotional intelligence (EI) in this association. This cross-sectional study was conducted between June and December of 2019. Participants were from seven medical schools in Lebanon. Higher extraversion (B = −0.11), higher neuroticism (B = −0.28) and higher emotional intelligence (B = −0.03) were significantly associated with lower depression. Higher neuroticism (B = −0.29) and higher emotional intelligence (B = −0.03) were significantly associated with lower anxiety. Higher openness to experience (B = 0.07) and higher agreeableness (B = 0.08) were significantly associated with higher stress, whereas higher neuroticism (B = −0.05) was associated with lower stress. EI mediated the association between extraversion and depression and openness to experience and depression. EI mediated the association between extraversion and anxiety and openness to experience and anxiety. The results of this study were different from those previously cited in the literature. This could be secondary to the mediating role of emotional intelligence. This study consequently opens up the possibility of new studies highlighting the role of emotional intelligence in the possible preservation of medical students’ mental health.

## 1. Introduction

Medical education is one of the most stressful and challenging education programs due to a large workload, time commitment and clinical environment pressure leading to a higher risk of psychological distress such as depression, anxiety and stress [1].

Depression is a serious medical illness that can decrease a person’s ability to function [2,3]. Depressed medical students may present with a decrease in educational performance, hopelessness and excessive sadness [3,4]. The prevalence of depression among medical students worldwide was estimated at 27.2% [3,5], with even higher rates found among Lebanese students (34.4%) [6]. Anxiety refers to “multiple mental and physiological phenomena, including a person’s conscious state of worry over a future unwanted event, or fear of an actual situation” [7,8]. Similar to depression, anxiety is highly prevalent among medical students—partially related to academic workload—and its global range varies between 29.2% and 38.7% [9,10]. Stress is a reaction experienced by the body, requiring mental, physical or psychological adjustment [11]. Medical students face high levels of stress that can be secondary to the huge amount of medical knowledge, lack of break time, and repetitive examination in highly competitive environment [12]. Stress among medical students is highly prevalent, with a variable range among countries varying from 66.4% in Lebanon [13] to 89.64% in India [14]. Academic stress among medical students is linked to poorer quality of life and higher rates of depression and anxiety symptoms [15].

Other than academic stress, the personality traits of medical students can play a major role in depression, anxiety, and stress [15,16]. Personality traits can be divided into five categories [15]: openness, conscientiousness, extraversion, agreeableness and neuroticism. People with openness tend to be more open to adventure and new findings, whereas conscientious persons tend to be more organized and have high sense of duty. Extroverts are persons who radiate energy, are cheerful, sociable and enjoy social interaction. Compassionate, helpful and kind people are described as agreeable. Finally, individuals with a high degree of neuroticism are worrisome persons and tend to fall more easily into depression, whereas those with a low degree of neuroticism tend to be more emotionally stable [17].

Besides personality traits, emotional intelligence was described to play an important role in medical students’ psychological wellbeing [1]. Emotional intelligence is defined as “the ability to control one’s own and others’ feelings and emotions, discriminate among them and use this information to guide one’s thinking and actions” [17]. High communication skills, self-awareness, flexibility, the capacity to handle new situations, empathy, and stress tolerance are qualities emotionally intelligent people have. These qualities tend to be a key to success in the medical field given the high-chasing technologies and workload demands [17]. Emotional intelligence was found to be a protective factor against stress in medical students [18]. In this context, emotional intelligence can have a mediating role, protecting against stress development and psychological distress in medical students as it gives a person a higher capability of dealing with stressful situations and coping with negative reactions [19].

To the knowledge of our group, this study is the first to assess the relationship between personality traits and depression, anxiety and stress among Lebanese medical students and to evaluate the mediating role of emotional intelligence in this association. In fact, the variables discussed in this study have been shown to affect the academic performance and quality of life of medical students [7,20]. The present study highlights the possible role of emotional intelligence in preventing anxiety, stress and depression in a population that is highly exposed to stressful events and in improving the academic performance of Lebanese medical students [9,20].

## 2. Materials and Methods

### 2.1. Study Design

This cross-sectional study took place between June and December of 2019. Our sample was selected from the seven national faculties in Lebanon. Inclusion criteria were as follows: students must have been of legal age (over 18 years) and enrolled in a full-time general medicine program. Students who filled out an online or paper questionnaire were included, reaching a total of 295 students. Informed consent was taken from all students who filled out the questionnaire without any compensation. Given that all seven of the national faculties of medicine require a prerequisite of good English knowledge, the questionnaire was filled out in English. A pilot study was used to assess questionnaire clarity prior to data collection. Pilot test-related data was not entered in the final database. This paper shares the same methodology used in a previous study [21].

### 2.2. Sample Size Calculation

The minimal sample size was set at 288 according to the Epi info program with an acceptable margin of error of 5% and an expected variance of depression among medical students of 27.2% worldwide [10] for 5531 general medicine students in Lebanon.

### 2.3. Questionnaire and Variables

The first part of the questionnaire consisted of sociodemographic characteristics such as age, gender, university and medical history. The second part included the following measures.

#### 2.3.1. Patient Health Questionnaire (PHQ-9)

The PHQ-9 is a nine-item self-report scale used to assess and monitor depression severity [22]. Higher scores reflect more severe depression (in this study, αCronbach = 0.911).

#### 2.3.2. Generalized Anxiety Disorder (GAD-7)

The GAD-7 is a seven-item self-report scale used to measure the severity of generalized anxiety disorders [23]. Higher scores indicate more severe anxiety (in this study, αCronbach = 0.923). Both the PHQ-9 and GAD-7 scales are validated in Lebanon [24].

#### 2.3.3. Perceived Stress Scale (PSS-4)

The PSS-4 measures the degree to which situations in one’s life over the past month are appraised as stressful [25]. Higher scores indicate a higher degree and longer duration of self-perceived stress (in this study, αCronbach = 0.628).

#### 2.3.4. The Big Five Personality Test (BFI)

Personality traits were assessed using the BFI, which includes the five broad personality traits: openness to experience (in this study, αCronbach = 0.718), conscientiousness (αCronbach = 0.640), extraversion (αCronbach = 0.880), agreeableness (αCronbach = 0.668) and neuroticism (αCronbach = 0.761). It includes 60 items, scored on a 5-point Likert-type scale [26]. A higher score on each trait indicates a higher presence of that personality trait.

#### 2.3.5. The Quick Emotional Intelligence Self-Assessment Scale

The Quick Emotional Intelligence Self-Assessment Scale includes four sections related to EI facets: self-management, self-awareness, social awareness and relationship management/social skills [27]. Each section includes ten questions, which are reported on a 5-point Likert-type scale. A higher EI was illustrated by higher total scores (in this study, αCronbach = 0.950).

### 2.4. Statistical Analysis

The Statistical Package for Social Science (SPSS) version 23 (IBM Corp., Armonk, NY, USA) was used to conduct the statistical analysis. There were no missing data since all questions were required in the form. The normality of distribution of the depression/anxiety/stress scores were confirmed via a calculation of the skewness and kurtosis; values for asymmetry and kurtosis between −1 and +1 were considered acceptable in order to prove normal univariate distribution [28]. The Student t and ANOVA tests were used to assess the association between the dependent variables and dichotomous/categorical variables, respectively. The correlation between two continuous variables was determined using the Pearson test. Forward linear regressions were conducted, taking depression, anxiety and stress as dependent variables. The PROCESS v.3.4 Model 4 was used to conduct the mediation analysis. Three pathways were calculated: (a) from the independent variable (each personality trait) to the mediator (emotional intelligence), (b) from the mediator (emotional intelligence) to the dependent variable (depression/anxiety/stress) and (c) from the independent variable (each personality trait) to the dependent one (depression/anxiety/stress). The mediation was considered significant if the Bootstrap confidence interval of the indirect effect did not pass by zero. All variables that showed significance in the bivariate analysis were entered in the linear regression and mediation model as independent variables. A value of *p* < 0.05 was considered statistically significant. Cronbach’s alpha was used to assess the reliability of scales and subscales.

## 3. Results

### 3.1. Sociodemographic and Other Characteristics of the Participants

The mean age of the participants was 22.41 ± 2.20 years, with 166 (56.1%) females. Other characteristics of the participants are summarized in Table 1.

### 3.2. Bivariate Analysis

The results of the bivariate analysis of factors associated with depression, anxiety and stress are summarized in Table 2 and Table 3. A higher mean depression score was found in students with low income compared to those with an intermediate and high income. A higher mean anxiety score was significantly found in females compared to males and in students with low income compared to those with an intermediate and high income. A higher mean stress score was found in students from Mount Lebanon compared to all other governorates. Furthermore, higher anxiety, stress and openness to experience were found to be significantly correlated with higher depression, whereas higher extraversion and neuroticism were significantly associated with lower depression. Higher stress and openness to experience were found to be significantly associated with higher anxiety, whereas higher extraversion and neuroticism were significantly associated with lower anxiety. Finally, higher age, agreeableness, conscientiousness, openness to experience and emotional intelligence were significantly associated with higher stress, whereas higher extraversion and neuroticism were significantly associated with lower stress.

### 3.3. Multivariable Analysis

Higher stress (B = 0.48) was significantly associated with higher depression, whereas higher extraversion (B = −0.11), higher neuroticism (B = −0.28) and higher emotional intelligence (B = −0.03) were significantly associated with lower depression (Table 4, Model 1).

Higher stress (B = 0.44) was significantly associated with higher anxiety, whereas higher neuroticism (B = −0.29) and higher emotional intelligence (B = −0.03) were significantly associated with lower anxiety (Table 4, Model 2).

Higher openness to experience (B = 0.07) and higher agreeableness (B = 0.08) were significantly associated with higher stress, whereas higher neuroticism (B = −0.05) was associated with lower stress (Table 4, Model 3). Notably, emotional intelligence was not significantly associated with stress.

### 3.4. Mediation Analysis

**Figure 1:**
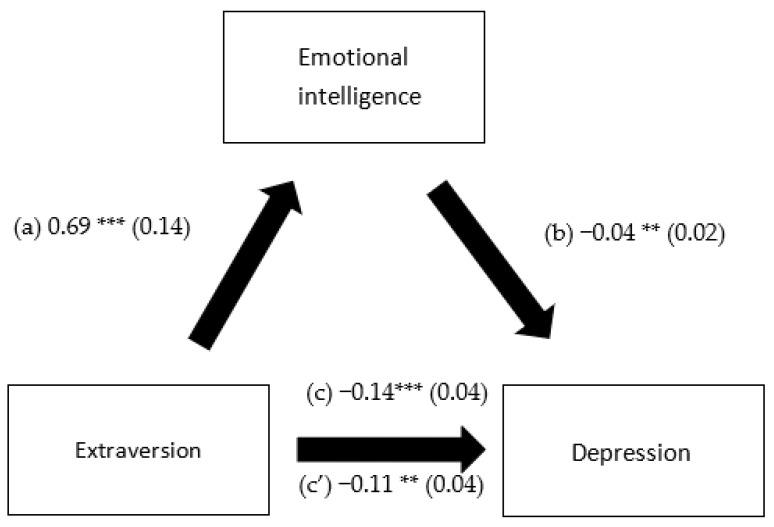
(a) Relation between extraversion and emotional intelligence (R^2^ = 0.294); (b) relation between emotional intelligence and depression (R^2^ = 0.284); (c) total effect of the relation between extraversion and depression (R^2^ = 0.270); and (c’) direct effect of the relation between extraversion and depression. Numbers are displayed as regression coefficients (standard error). ** *p* < 0.01; *** *p* < 0.001.

**Figure 2:**
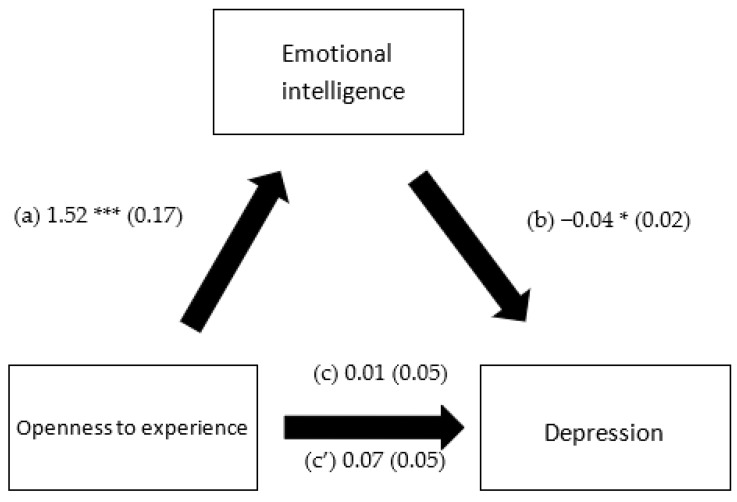
(a) Relation between openness to experience and emotional intelligence (R^2^ = 0.294); (b) relation between emotional intelligence and depression (R^2^ = 0.284); (c) total effect of the relation between openness to experience and depression (R^2^ = 0.270); and (c’) direct effect of the relation between openness to experience and depression. Numbers are displayed as regression coefficients (standard error). * *p* < 0.05; *** *p* < 0.001.

**Figure 3:**
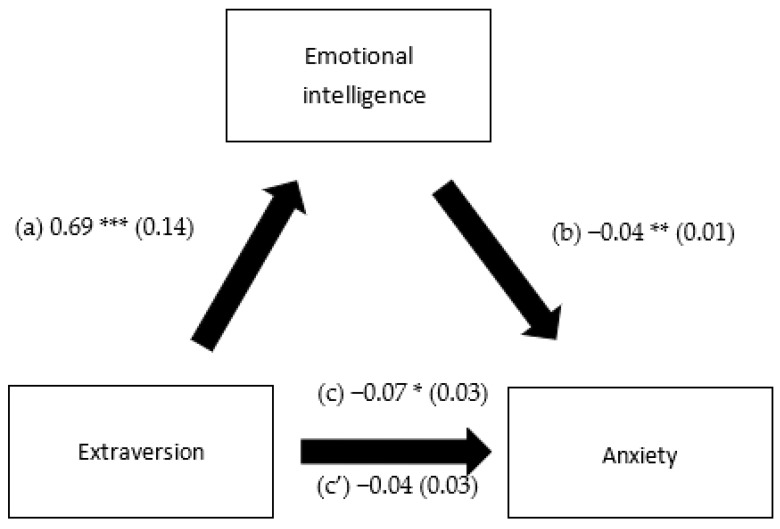
(a) Relation between extraversion and emotional intelligence (R^2^ = 0.294); (b) relation between emotional intelligence and anxiety (R^2^ = 0.344); (c) total effect of the relation between extraversion and anxiety (R^2^ = 0.325); and (c’) direct effect of the relation between extraversion and anxiety. Numbers are displayed as regression coefficients (standard error). * *p* < 0.05; ** *p* < 0.01; *** *p* < 0.001.

**Figure 4:**
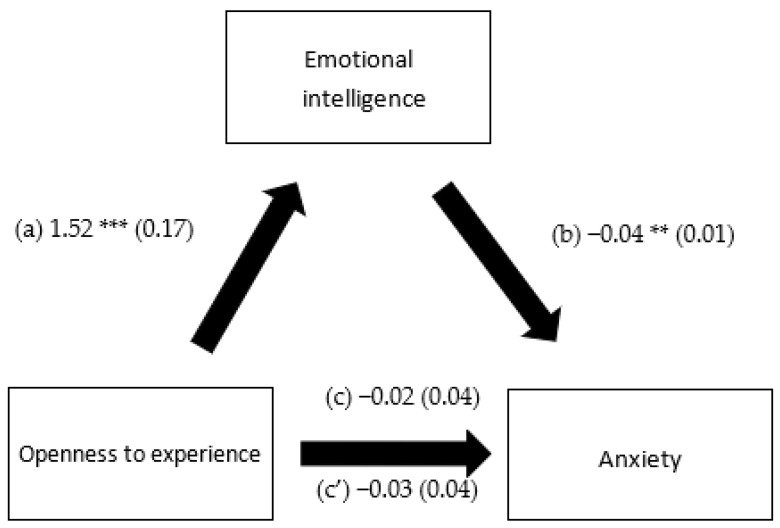
(a) Relation between openness to experience and emotional intelligence (R^2^ = 0.294); (b) relation between emotional intelligence and anxiety (R^2^ = 0.284); (c) total effect of the relation between openness to experience and anxiety (R^2^ = 0.270); and (c’) direct effect of the relation between openness to experience and anxiety. Numbers are displayed as regression coefficients (standard error). ** *p* < *0*.01; *** *p* < *0*.001.

EI mediated the association between extraversion and depression, openness to experience and depression, extraversion and anxiety and openness to experience and anxiety. However, it did not mediate the association between any of the personality traits and stress (Table 5). Higher extraversion and openness to experience were significantly associated with more EI. Higher EI was significantly associated with lower depression and anxiety. Finally, higher extraversion was significantly and directly associated with less depression (Figure 1, Figure 2, Figure 3 and Figure 4).

## 4. Discussion

Higher extraversion and neuroticism were significantly associated with lower depression. Higher neuroticism was significantly associated with lower anxiety. Higher openness to experience and agreeableness were significantly associated with higher stress, whereas higher neuroticism was associated with lower stress. Finally, EI mediated the association between extraversion and depression, openness to experience and depression, extraversion and anxiety and openness to experience and anxiety.

### 4.1. Personality and Psychological Disorders (Depression, Anxiety and Stress)

In this study, we found that higher extraversion was associated with lower rates of depression, corroborating the findings of previous studies [15,29]. Extraversion was described in the literature as a protective factor for stress-related disorders [15]. Extraverts always seek new experiences, cope well with new challenges and situations and can be less affected by the large loads of new information and challenges medical students face. Hence, extroverts are less affected by such challenges and consequently develop less anxiety and depression [30].

Having a neurotic personality trait was described to have the strongest positive correlation with all psychological health problems in medical students during stressful periods [30]. In fact, neuroticism is characterized by high nervousness and emotional instability [31]. All these factors can predispose medical students to higher stress [31]. However, in our study and in opposition to many published studies [15,29], neuroticism was associated with lower levels of depression, anxiety and stress. This result can be secondary to many confounding factors that can be present in medical students with neuroticism but were not assessed in this study (such as emotional regulation, self-esteem, social support, etc.).

In this study, we found higher openness to experience to be associated with higher levels of stress. In fact, people with high openness to experience tend to be intellectually curious with a constant urge for new experiences and adventures. Due to the large workload they face, medical students have to choose between their private lives in parallel with their medical studies or their success in medical school [32]. In fact, a study performed on German medical students showed that more than one-third of medical students reported having no time to continue personal activities and were forced to neglect all activities except their medical studies. This statement can explain why medical students who are adventure seekers and have high openness to experience perceive higher levels of stress [33].

Furthermore, our study found that agreeableness was associated with higher levels of stress in medical students. However, most studies in the literature found that agreeableness can be a protective factor against stress predisposition [34]. Our finding can be explained by the fact that agreeableness gives medical students a constant need to help others; they are trusting people, straightforward, cooperative and modest [35]. However, the highly competitive environment Lebanese medical students must survive in can make cooperative and helpful, agreeable individuals feel uncomfortable and more stressed [13,36]. These reasons can fall behind the higher levels of stress found in our study. Additionally, our results came in concordance with the findings of Getzmann et al. (2021) who studied the impact of personality traits on emotional stress caused by the coronavirus disease 19 [37]. In addition, other non-assessed confounding factors can be present and were not assessed in the present study (such as low self-esteem, emotion dysregulation, lack of communication, social support, etc.).

### 4.2. Mediation between Personality Traits, EI and Psychological Disorders

This study found that emotional intelligence was associated with lower depression and anxiety. These results are similar to those found in Iranian physicians, where emotional intelligence was found to be a predictor of lower levels of stress, anxiety and depression [38]. In fact, it was previously described that emotional intelligence gives medical students the ability to overcome stressful events by providing them with the capacity to understand their own as well as others’ emotions, consequently allowing them to make better life choices. In addition, they will have a better control on their emotional state and can know when to express their feelings [38,39].

A study conducted on medical students showed that many aspects of emotional intelligence can play a mediating role for a better psychological health [21]. For instance, emotional fortitude, emotional control and emotional maturity were found to have a weak-to-moderate correlation with medical students’ mental health; this correlation can be explained by the fact that higher emotional intelligence was described to be associated with better emotional wellbeing. This statement can explain the mediating effect of emotional intelligence and its protective role against depression and anxiety in medical students [19].

The relationship between personality traits and emotional intelligence has long been investigated. Emotionally intelligent people are understanding and have a high capacity for controlling their own as well as others’ emotions. In addition, extraverts tend to be more open to experience and consequently have higher emotional intelligence traits [29]. Extraverted medical students were found to be happier, to have more positive emotion dispositions [40] and to have more emotional intelligence than others do, and consequently are less prone to depression/anxiety [41]. Similarly, we found that extraverted medical students had higher levels of emotional intelligence and lower levels of depression/anxiety, with EI mediating this association. This is in line with a recent study conducted among Healthcare Workers Saudi Arabia [42].

Furthermore, our study showed that EI mediated the association between openness to experience and depression/anxiety, in line with previous studies [42,43]. For instance, individuals with high scores of openness to experience use more communication to enhance positive emotions and regulate negative emotions [44]. In parallel with being open to new experiences, emotionally intelligent medical students know how to handle their own and others’ emotions and how to deal with emotions effectively, which leads to a better adaptation to the demands and challenges of the medical program. This association (between openness to experience and emotional intelligence) is able to support the medical students in experiencing more academic satisfaction, thus achieving better mental health [8].

### 4.3. Clinical Implications

Our study highlights the important role of emotional intelligence in medical students and its mediating effect on mental health. Consequently, fostering emotional intelligence in medical students may possibly have a protective role against psychological distress, and may be a key factor that helps medical students endure stressful challenges. Further longitudinal studies are needed to assess the role of emotional intelligence in the psychological wellbeing of medical students and its effect on academic performance.

### 4.4. Limitations

Our study has many limitations. A residual confounding bias may be present since not all confounding factors associated with mental illness were taken into consideration in this study (e.g., emotion regulation, emotion difficulties, alexithymia, etc.). This may explain differences in results compared to the literature. In addition, given the fact that it is a cross-sectional study, we cannot assess a true causal relationship between emotional intelligence, personality traits and psychological disorders. The results cannot be generalized to all medical students since not all medical schools were equally represented. An information bias is also possible, as in all cross-sectional studies participants might under- or over-rate their answers to some questions. In addition, the presence of mental illness was self-reported by the students and not evaluated by a mental health specialist.

## 5. Conclusions

Our study demonstrated the association between some personality traits and mental illness. The results of this study were different from those previously cited in the literature; this could be secondary to the mediating role of emotional intelligence. Emotional intelligence training programs are nowadays frequently used by many employers to improve employees’ productivity and well-being [36]. This study consequently opens up the possibility for new studies to highlight the role of emotional intelligence as a protective factor against depression, anxiety and stress. The present study may serve as a valuable tool for medical schools’ administrations to refine their selection criteria and to develop targeted measures to foster emotional intelligence among their students.

## Figures and Tables

**Table 1:** Sociodemographic and other characteristics of the participants (*n* = 296).

Variable	*n* (%)
**Gender**	
Male	130 (43.9%)
Female	166 (56.1%)
**Governorate**	
Beirut	28 (9.5%)
Mount Lebanon	124 (41.9%)
North	74 (25.0%)
South	37 (12.5%)
Bekaa	33 (11.1%)
**Monthly income**	
Low (<1000 USD)	21 (7.1%)
Intermediate (1000–2000 USD)	133 (44.9%)
High (>2000 USD)	142 (48.0%)
**University**	
American University of Beirut	20 (6.8%)
Beirut Arab University	13 (4.4%)
Holy Spirit University of Kaslik	133 (44.9%)
Lebanese American University	17 (5.7%)
Lebanese University	80 (27.0%)
Saint Joseph University	14 (4.7%)
University of Balamand	19 (6.4%)

**Table 2:** Bivariate analysis of factors associated with the depression, anxiety and stress scores.

Variable	Depression	Anxiety	Stress
**Gender**			
Male	8.24 ± 6.67	5.81 ± 5.33	14.39 ± 6.45
Female	8.34 ± 6.49	6.96 ± 5.34	15.67 ± 5.82
*p*	0.898	0.065	0.076
Effect size	0.015	0.215	0.208
**Monthly income**			
Low (<1000$)	9.81 ± 6.52	7.24 ± 5.38	16.61 ± 5.14
Intermediate (1000–2000$)	9.22 ± 6.82	7.45 ± 5.53	15.28 ± 5.80
High (>2000$)	7.20 ± 6.17	5.41 ± 5.01	14.72 ± 6.55
*p*	**0.021**	**0.005**	0.384
Effect size	0.163	0.191	0.081

Numbers in bold indicate significant *p* values.

**Table 3:** Bivariate analysis of continuous variables associated with the mental health scores.

Variable	Depression	Anxiety	Stress
Depression	1		
Anxiety	r = 0.655; ***p* < 0.001**	1	
Stress	r = 0.359; ***p* < 0.001**	r = 0.392; ***p* < 0.001**	1
Age	r = −0.034; *p* = 0.562	r = −0.02; *p* = 0.728	r = 0.113; *p* = 0.051
Extraversion	r = −0.310; ***p* < 0.001**	r = −0.267; ***p* < 0.001**	r = −0.105; *p* = 0.07
Agreeableness	r = 0.021; *p* = 0.721	r = 0.062; *p* = 0.290	r = 0.257; ***p* < 0.001**
Conscientiousness	r = −0.019; *p* = 0.751	r = 0.036; *p* = 0.542	r = 0.1; *p* = 0.086
Neuroticism	r = −0.479; ***p* < 0.001**	r = −0.549; ***p* < 0.001**	r = −0.257; ***p* < 0.001**
Openness to experience	r = 0.181; ***p* = 0.002**	r = 0.165; ***p* = 0.004**	r = 0.269; ***p* < 0.001**
Emotional intelligence	r = −0.072; *p* = 0.218	r = −0.078; *p* = 0.181	r = 0.05; *p* = 0.395

r = Pearson correlation coefficients; numbers in bold indicate significant *p* values.

**Table 4:** Multivariable analyses.

Model 1: Linear Regression Taking the Depression Score as the Dependent Variable.				
Variable	Unstandardized Beta	Standardized Beta	*p*	95% CI
Stress	0.48	0.24	**<0.001**	0.28–0.68
Extraversion	−0.14	−0.19	**0.006**	−0.18–−0.03
Neuroticism	−0.28	−0.38	**<0.001**	−0.36–−0.20
Emotional intelligence	−0.03	−0.13	**0.03**	−0.06–−0.003
**Model 2: Linear regression taking the anxiety score as the dependent variable.**				
**Variable**	**Unstandardized Beta**	**Standardized Beta**	***p***	**95% CI**
Stress	0.44	0.27	**<0.001**	0.29–0.60
Neuroticism	−0.29	−0.48	**<0.001**	−0.35–−0.23
Emotional intelligence	−0.03	−0.14	**0.011**	−0.05–−0.01
**Model 3: Linear regression taking the stress score as the dependent variable.**				
**Variable**	**Unstandardized Beta**	**Standardized Beta**	***p***	**95% CI**
Openness to experience	0.07	0.17	**0.013**	0.02–0.13
Agreeableness	0.08	0.19	**0.002**	0.03–0.13
Neuroticism	−0.05	−0.14	**0.025**	−0.10–−0.01
Emotional intelligence	−0.01	−0.09	0.206	−0.03–0.01

Numbers in bold indicate significant *p* values; CI = Confidence Interval; Variables entered in model 1: Income, Stress, Extraversion, Neuroticism, Openness to experience, Emotional intelligence; Variables entered in model 2: Gender Income, Stress, Extraversion, Neuroticism, Openness to experience, Emotional intelligence; Variables entered in model 3: Extraversion, Openness to experience, Emotional intelligence, Age, Agreeableness, Conscientiousness, Neuroticism.

**Table 5:** Mediation analyses results, taking each personality trait as an independent variable, emotional intelligence as a mediator and depression/anxiety/stress as dependent variables.

Mediator	Direct Effect			Indirect Effect		
	Beta	SE	*p*	Beta	Boot SE	Boot CI
**Model 1: Depression as the Dependent Variable**						
Extraversion	−0.11	0.04	0.005	−0.03	0.01	−0.06; −0.01 *
Neuroticism	−0.31	0.04	<0.001	−0.01	0.01	−0.01; 0.02
Openness to experience	0.07	0.05	0.173	−0.06	0.02	−0.10; −0.01 *
**Model 2: Anxiety as the dependent variable**						
Extraversion	−0.04	0.03	0.158	−0.02	0.01	−0.05; −0.01 *
Neuroticism	−0.31	0.03	<0.001	0.005	0.01	−0.01; 0.02
Openness to experience	0.03	0.04	0.463	−0.05	0.02	−0.09; −0.02 *
**Model 3: Stress as the dependent variable**						
Agreeableness	0.08	0.03	0.001	0.001	0.004	−0.01; 0.01
Neuroticism	−0.06	0.02	0.006	−0.001	0.003	−0.01; 0.01
Openness to experience	0.08	0.03	0.006	−0.02	0.02	−0.05; 0.02

* indicates a significant mediation model.

## Data Availability

The authors do not have the right to share any data information publicly as per their institutions’ policies. Information can be shared upon reasonable request from the corresponding author (S.H.).
